# SpykProps: an imaging pipeline to quantify architecture in unilateral grass inflorescences

**DOI:** 10.1186/s13007-023-01104-z

**Published:** 2023-11-13

**Authors:** Joan Barreto Ortiz, Candice N. Hirsch, Nancy Jo Ehlke, Eric Watkins

**Affiliations:** 1 Department of Agronomy and Plant Genetics, University of Minnesota, St. Paul, MN 55108 USA; 2 Department of Horticultural Science, University of Minnesota, St. Paul, MN 55108 USA

**Keywords:** Inflorescence architecture, Latent phenotypes, Image analysis, High-throughput phenotyping, Machine learning

## Abstract

**Background:**

Inflorescence properties such length, spikelet number, and their spatial distribution across the rachis, are fundamental indicators of seed productivity in grasses and have been a target of selection throughout domestication and crop improvement. However, quantifying such complex morphology is laborious, time-consuming, and commonly limited to human-perceived traits. These limitations can be exacerbated by unfavorable trait correlations between inflorescence architecture and seed yield that can be unconsciously selected for. Computer vision offers an alternative to conventional phenotyping, enabling higher throughput and reducing subjectivity. These approaches provide valuable insights into the determinants of seed yield, and thus, aid breeding decisions.

**Results:**

Here, we described SpykProps, an inexpensive Python-based imaging system to quantify morphological properties in unilateral inflorescences, that was developed and tested on images of perennial grass (*Lolium perenne* L.) spikes. SpykProps is able to rapidly and accurately identify spikes (RMSE < 1), estimate their length (R^2^ = 0.96), and number of spikelets (R^2^ = 0.61). It also quantifies color and shape from hundreds of interacting descriptors that are accurate predictors of architectural and agronomic traits such as seed yield potential (R^2^ = 0.94), rachis weight (R^2^ = 0.83), and seed shattering (R^2^ = 0.85).

**Conclusions:**

SpykProps is an open-source platform to characterize inflorescence architecture in a wide range of grasses. This imaging tool generates conventional and latent traits that can be used to better characterize developmental and agronomic traits associated with inflorescence architecture, and has applications in fields that include breeding, physiology, evolution, and development biology.

**Supplementary Information:**

The online version contains supplementary material available at 10.1186/s13007-023-01104-z.

## Background

Inflorescences are flower-bearing structures composed of multidimensional traits that are shaped by both natural and artificial selection. These selective pressures and developmental changes over variable climates resulted in plants with highly complex inflorescence architectures [31]. Hidden in this complexity are the relationships between micro and macroscopic inflorescence traits that attract biotic and abiotic dispersers and allow gene flow across space and time [5, 33]. For example, short-distance dispersal relies on shattering, a programmed disarticulation of inflorescence structures due to a microscopic abscission formation, as well as insects. In contrast, long-distance dispersal is associated with wind, water, and vertebrates [36], and thus depends on macroscopic or visible traits. For humans, selecting on inflorescence properties that reduced dispersal without affecting fitness was paramount to plant domestication and to continued efforts to increase grain yield and food supply.

Spikes, panicles, and seed heads, all refer to grass inflorescences, the morphology of which contributes to seed yield potential in grasses [1]. In grasses that are not fully domesticated, poor seed yield is commonly attributed to spike properties that facilitate dispersion such as shattering and asynchronous flowering and ripening. The rate at which such traits can be improved through breeding depends on their genetic architecture as well as our ability to properly quantify and select the traits that associate with them [47, 48]. This is a major challenge for yield increase because the myriad of traits comprising inflorescence architecture are often pleiotropic [35] and have complex genetic correlations [30] that may result in indirect selection for unfavorable traits [13, 22, 34, 37, 43], decreasing breeding efficiency. Furthermore, selection on spike properties is time consuming, low-throughput, costly, and restricted to traits that can be perceived or measured.

Because plant phenotypes are infinite over the lifetime of an organism, phenotyping at a single point in time provides the best estimates of their underlying genetic architecture at a given environment and developmental stage [10, 14]. The development of high-throughput phenotyping (HTP) systems has contributed to automating the measurement of complex traits and estimating this infinite phenotypic space [44]. High-throughput phenotyping systems to characterize inflorescence architecture are less common. Image-based tools such as P-TRAP [3] and PANorama [11] have been developed to estimate properties such as length, spikelet number, and branching patterns in rice. Similar methods exist for *Zea mays* [16], *Sorghum bicolor *[46], *Triticum* [20, 27], and *Avena sativa* [6]. While these tools have been useful in the breeding (Hamsa Poorna [19] and studying the genetic control of inflorescence morphology [12, 32], they focus on conventional traits such as spike length and spikelet number. While their interpretability are a key advantage for plant breeding, the exclusive focus on these conventional traits impedes exploring the effect of subtle traits, i.e., latent traits, as the interacting components of multidimensional inflorescence architecture [10, 14].

Here we describe SpykProps, a tool that, unlike other imaging approaches, allows one to derive latent phenotypes from a myriad of interacting descriptors of color and shape that focus on the inflorescence as a multidimensional phenotype. In addition, our tool can accurately measure conventional traits such as spike length, area, and spikelet number, and does not require proprietary software or sophisticated imaging hardware. This system was developed and tested in perennial ryegrass (*Lolium perenne* L.), a multifunctional crop that due to its recent domestication for seed production suffers from yield reductions from wild spike architectural traits. Nonetheless, SpykProps can be used to study other species with two-ranked unilateral, i.e., flat inflorescences [21] in fields including breeding, physiology, evolutionary biology, and developmental biology.

## Results

**Fig. 1:**
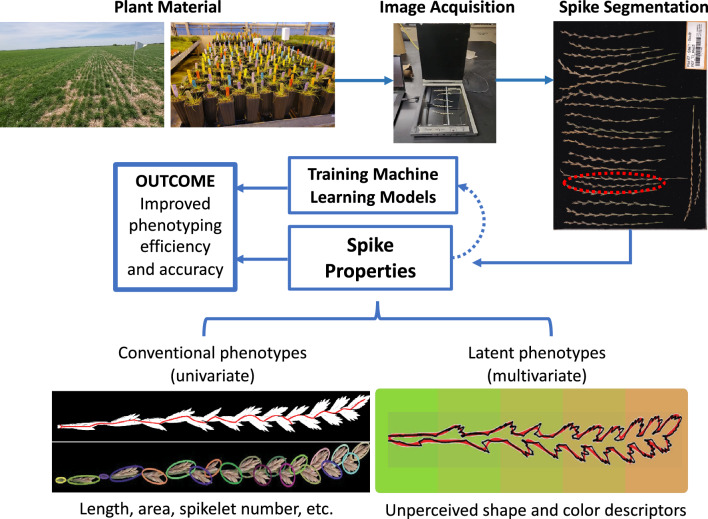
Overview of the SpykProps pipeline in the context of agronomic or breeding research. Spikes from field or control conditions, can be imaged in situ or harvested for further processing in a dry lab. SpykProps segments spikes and quantifies color and shape, using univariate and multivariate descriptors. This enables rapid, holistic, and less biased characterization of multidimensional spike architecture variations, offering researchers improved phenotyping tools, especially when combined with machine learning techniques

Our goal was to develop an inexpensive high-throughput system capable of quantifying spike architecture in species with a two-ranked unilateral inflorescence, such as perennial ryegrass. Accordingly, we developed SpykProps, an imaging pipeline written in Python to characterize color and shape-based properties of the spike and spikelets. This system is aimed to be implemented in breeding and research programs seeking to unveil subtle relationships between architectural and dispersal traits affecting yield, using RGB images (Fig. 1). The pipeline was built using 8-bit color images obtained with flatbed scanners at 600 dpi that have a black non-reflective background. However, the functions in the program have flexibility to process images of variable size, resolution, and background color. SpykProps is an open-source program that can be accessed from https://github.com/joanmanbar/SpykProps along with detailed instructions to analyze single spikes using a Python integrated development environment, or to automate it on a set of images using Bash and the *SpykBatch.py* function. In either run mode, images are imported and parameters can be set to export datasheets with hundreds of color and shape descriptors per spike and spikelet, along with images of the segmented structures for visual validation.

### Spike segmentation

The first step in the automated pipeline is to identify spikes in the image and separate them from background and other debris. This requires a threshold that can either be set on a channel from the red-green-blue (RGB) value representation, or can be estimated automatically using the Otsu algorithm [29]. This algorithm uses the pixel intensity histogram in gray scale to maximize separability between classes, resulting in a stringent threshold when using dark backgrounds and therefore requires a scaling factor. In Spykprops, spikes are detected using the *spike_segm* function calling on either the *channel_thresh* option for manual RGB thresholding or the *OtsuScaling* option for automated segmentation. At this step, users can also opt to rescale the original images by a factor (*rescale_rgb*), resulting in fewer pixels and detail to process, reducing computational time. The *spike_segm* function produces segmented spikes in individual output images with different representations including, RGB, CIELAB (L*a*b*), and hue-saturation-value (HSV). Using multiple spaces provides a broader range of colors which increases the ability to discriminate across spikes with similar color profiles. Selecting a proper method and threshold that accurately segments the spike is essential since the binary mask that results from this step directly affects the quality of the color and shape descriptors.

**Fig. 2:**
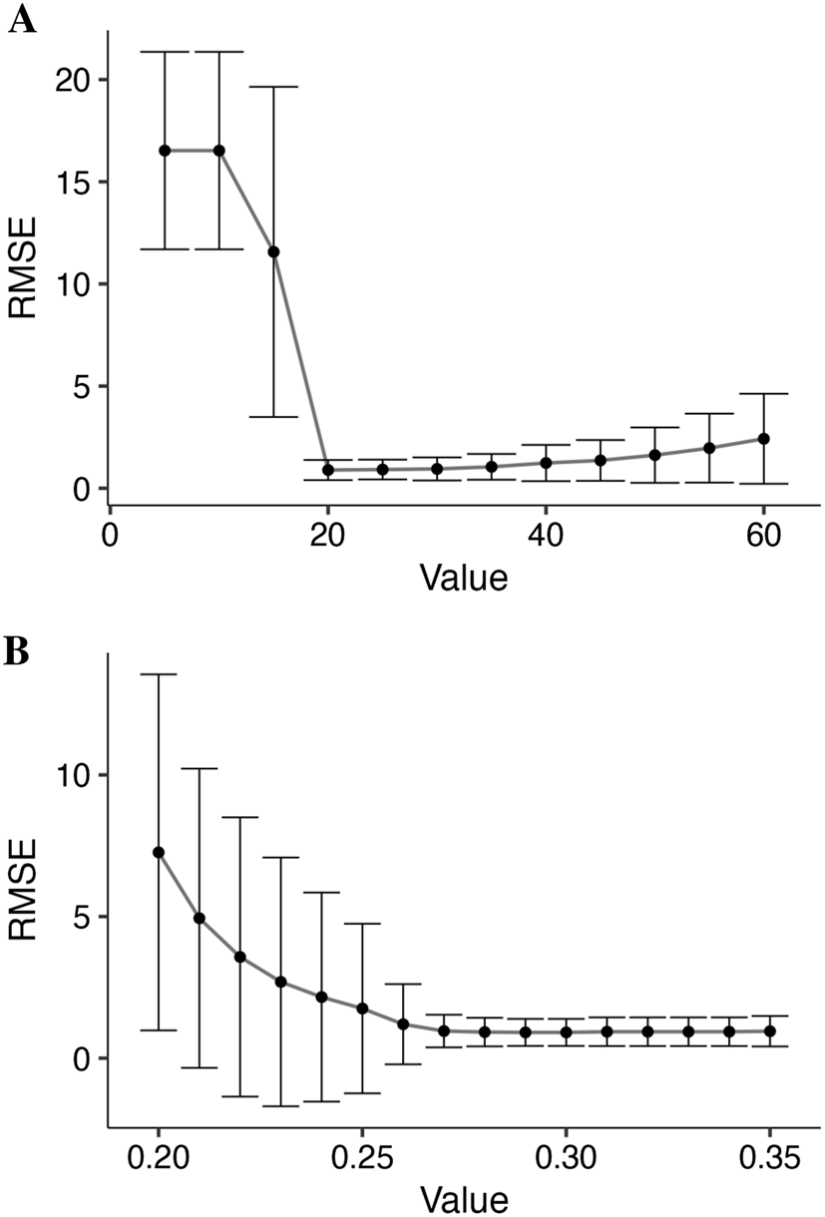
Spike segmentation methods included in SpykProps. **A** Segmentation based on input threshold for the red channel. **B** Otsu segmentation scaled by a factor. RMSE (y-axis) is the root mean squared error. Error bars represent the standard deviation

To determine optimal thresholds for accurately segmenting spikes in our images, we evaluated different thresholds on the red channel as well as scaling factors for the Otsu algorithm (Fig. 2). Specifically, 116 images containing 2743 spikes were run through SpkyPros rescaled at 50% on each axis and were also manually segmented. Pixel values between 20 and 35 on the red channel and scaling the Otsu threshold by 28–30% had a RMSE < 1, i.e., an average lower than one misdetected spike (0.03%). The optimal threshold value on the red channel was 20 (RMSE = 0.89 ± 0.49) and the best scaling factor for the Otsu threshold was 29% (RMSE = 0.91 ± 0.48). The time to detect the 2743 spikes and generate binary masks for all tested thresholds across both methods was 184 min, with an average of 0.14 s per spike and threshold. It is important to note that optimal thresholds may vary according to image resolution, size, light conditions, and the color and texture of the image background. These features of the image may also impact processing time and accuracy. As such, we included a Python script (*SpikeSeg_Batch.py*) within SpykProps to assist users in determining the method and threshold that is most optimized to their image properties.

### Spike length

**Fig. 3:**
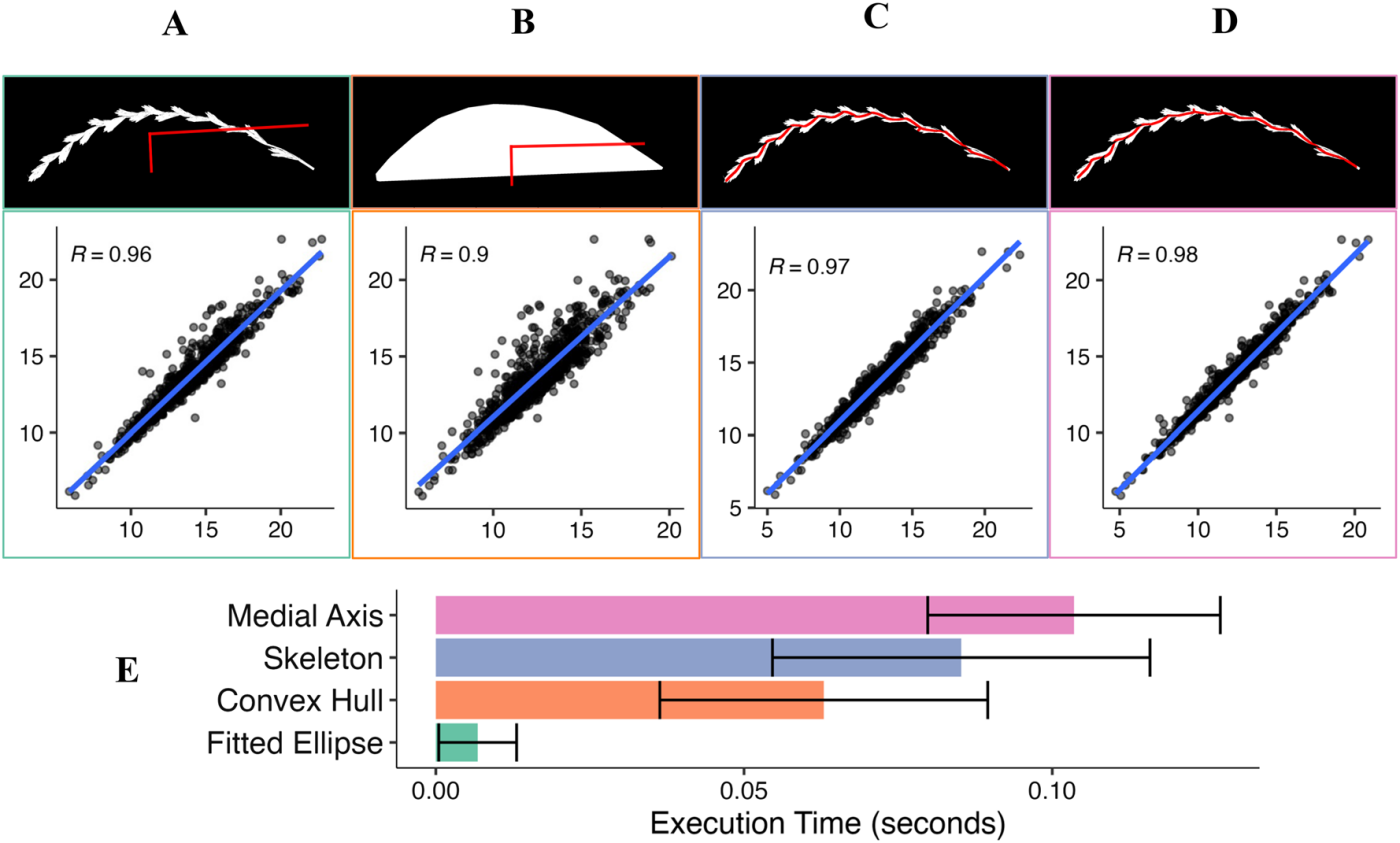
Spike length approximation methods included in SpykProps. Figures **A**–**D** show in the top: a binary mask with an overlayed red line indicating the approximated length; and the bottom: the scatterplot with a regression line of predicted (x-axis) and observed (y-axis) lengths, with the Pearson correlation coefficient (R; p < 0.001). The spike length in **A** and **B** are approximated as twice the major axis (longest red line) of a fitted ellipse on the spike (**A**) or its convex hull (**B**). The spike length in **C** and **D** are determined by Zhang’s (**C**) or medial axis (**D**) skeletonization algorithms. (**E**) Average execution time per spike in seconds across methods with standard error calculated from execution time of 810 binary images

Spike length is a major determinant of spike architecture in the grasses [1, 28], and a key component of seed yield in perennial ryegrass [2]. However, measuring this trait in field or lab conditions can be tedious, time intensive, and low-throughput, making it an important trait for image based acquisition. Imaging strategies often approximate length from the major axis' distance of a geometric object such as an ellipse, fitted on the object of interest (Fig. 3A) or its convex hull (Fig. 3B). This strategy works well on straight spikes but has reduced accuracy with curved spike architectures. To account for this curved architecture that is commonly observed in perennial ryegrass, and many other species for which SpykProps could be applied, we implemented two additional methods that reduce the spike's binary mask to a single-pixel (thinning) after applying a low-pass filter (blurring). These methods include *skeleton*, which relies on Zhang and Suen’s [45] skeletonization algorithm (Fig. 3C), and *medial_axis*, which computes the medial axis transform of the spike (Van der Walt et al., n.d.) (Fig. 3D). Any or all of these four methods can be specified on the *spk_length* function which returns the estimated length, computing time, and in the case of the thinning methods an image of the spike’s binary mask with the overlay of the length (Fig. 3C, D).

To determine the accuracy of each of the length detection methods that are built into SpykProps, we compared predicted lengths to manual measurements of spike length obtained using ImageJ [15] for 810 segmented spikes. As predicted, thinning algorithms, i.e., medial axis (R^2^ = 0.96) and skeleton (R^2^ = 0.95) that can account for the curved nature of some spikes were more accurate in approximating spike length than using the major axis of a fitted ellipse on the binary spike (R^2^ = 0.92) or on its convex hull (R^2^ = 0.81) (Fig. 3). Nevertheless, there was a tradeoff between accuracy and execution time for the thinning algorithms (Fig. 3E). Using a fitted ellipse on the spike was on average 15 times faster than the medial axis method. The fastest method, fitted ellipse, was also less variable (0.007 ± 0.006 s) than medial axis (0.103 ± 0.023 s), convex hull (0.063 ± 0.027 s), and skeleton (0.086 ± 0.030 s). However, this variability in execution time is highly dependent on the proportion of irregular spikes in the set of images. Taken together, this analysis demonstrates that if curved spikes are common in a dataset, the medial axis is the ideal approach despite the computational cost. Otherwise, the length of a fitted ellipse on the spike will suffice to provide an accurate approximation of spike length.

### Spikelet segmentation

**Fig. 4:**
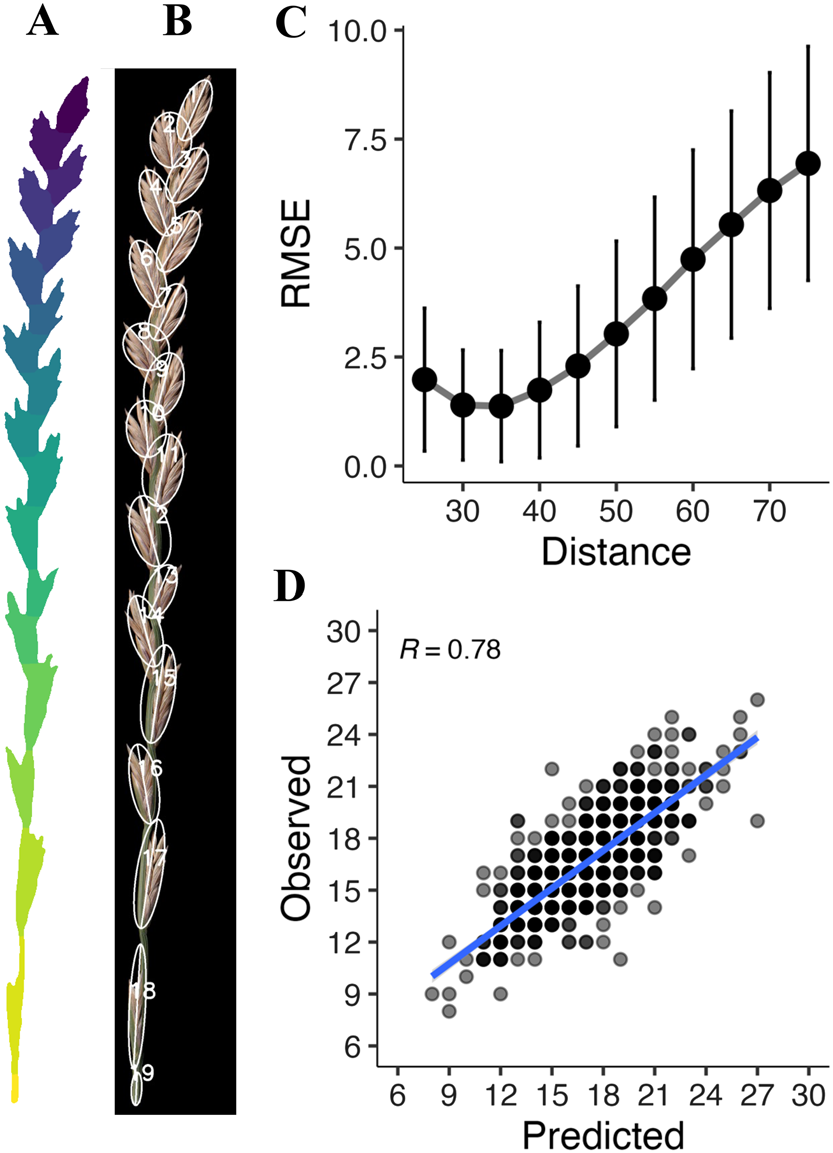
Assessment of the spikelet detection method included in SpykProps. **A**, **B** Output images from the spikelet_segm function where each color in **A** represents an approximated spikelet region, to which a numbered ellipse and its major axis line will be fitted (**B**). **C** Root mean squared error (RMSE) across minimum Euclidean distances in pixels expected between the center of a spikelet. **D** Scatterplots of the predicted (x-axis) versus observed (y-axis) number of spikelets per spike, with their Pearson correlation coefficient (R; p < 0.001)

In addition to spike length, the number of spikelets and distribution across the rachis are key components of seed productivity [21]. To segment individual spikelets, we compiled a series of algorithms in a single function (*spikelet_segm*) that returns spikelet count and generates a mask to quantify their morphology. The first steps involve adding a pad around the spike to facilitate morphological operations such as erosion and opening, which are performed using a cross-shaped kernel of 3 × 3 pixels. These convolutions are executed on a 10% rescaled version of the spike's mask to increase performance, before it is rescaled back to its original size to generate a mask of the Euclidean distances from each pixel to the nearest background pixel. The local peaks in the mask are calculated according to the expected minimum distance (in pixels) between spikelets and are used as markers for the watershed algorithm, which approximates the spikelets boundaries by treating the transformed distance as a topographic map flooded with water (Fig. 4A). Next, an ellipse is fitted on each spikelet contour and its major axis length is used to estimate the spikelet's angle (Fig. 4A) with respect to the top-left image corner. Spikelet detection accuracy primarily depends on the minimum distance expected between spikelets, which must be defined a priori. Such distance must be carefully established based on the type of misdetections and whether the misdetections can be easily filtered based on size (Additional file 1: Fig S2). Users can determine the best minimum distance for their inflorescences from a range of values in the *SpikeletSeg_batch.py* program, which also generates images of the detected spikelets as in Fig. 4A and a dataset with the number of detected spikelets, as well their ellipse’s area, length, and relative angle (Fig. 4B).

To assess the accuracy of our spikelet segmentation method, we visually counted the number of spikelets across 793 spikes and compared them with automated detection. The spikes were segmented using a minimum threshold of 25 in the red channel and a range of minimum distances from 25 to 75 pixels between spikelets. Our results indicated that values between 25 and 40 pixels missed or overestimated less than two spikelets per spike on average (Fig. 4C). We then selected a minimum distance of 25 pixels (1.98 ± 1.64 misdetections) to validate the spikelet count and found a strong Pearson correlation (r = 0.78) between the automated and manual spikelet count on 793 spikes (Fig. 4D). This indicates relatively high accuracy (R^2^ = 0.61) with potential for improvement using conditional or variable minimum expected distances between spikelets.

### Quantifying spike shape

The ubiquity of irregular shapes in plant morphology poses a challenge in accurately quantifying phenotypic variation. As a result, conventional phenotyping methods tend to prioritize interpretability and ease of measurement, leading to potential biases and a limited scope of the multidimensional phenotype [14, 25]. SpykProps addresses these challenges in two ways: using both geometric descriptors that are easy to interpret, and elliptical Fourier descriptors, which approach the overall spike shape from a multidimensional perspective.

Geometric descriptors are generated from the binary mask resulting in segmenting spikelets, also referred to as regions of interest (ROI). This step relies on the *regionprops* function from scikit-image (Van der Walt et al., n.d.), which generates over 20 possible properties; however, we only focused on those that are more relevant to spike architecture (Table 1). Nevertheless, additional variables can be retrieved with minor changes in the *SpikeDF* and *SpikeletDF* functions within SpykProps, based on specific researcher needs. These descriptors can be combined with spike length, number of spikelets, and other univariate traits, to derive more robust and uncorrelated phenotypes using dimensional reduction techniques.

**Table 1:** Geometric descriptors of spike and spikelet shape

Property	Description	Purpose
Area^a^	Number of pixels within the ROI^b^	Quantifies the ROI’s area
Eccentricity	Measure of elongation	Helps to identify curved spikes
Equivalent diameter	Diameter of a circle with same the area	Provides a latent measure of ROI's size and shape with reference to a circle
Extents	Ratio of the ROI’s bounding box area to the *Area*	Provides a latent measure of the ROI's spread or aperture
Ferets	Estimate of longest and shortest distance within the ROI’s outline	Useful to estimate width on non-curved ROI's. Unlike *Minor Axis*, it considers the overall shape rather than a fitted ellipse
Major axis	Length of the longest axes of an ellipse fitted on the ROI	Provides a fast estimate of length on non-curved ROI's
Minor axis	Length of the shortest axes of an ellipse fitted on the ROI	Provides a fast estimate of width on non-curved ROI's
Perimeter	Length of the ROI's outline	Provides a latent measure of the ROI's size
Solidity	Ratio of the *Area* to the ROI's convex hull's area	Provides a latent measure of shape

^a^Italic features refer to those described in the column Property

^b^Region of interest (ROI), specifically refers to a segmented spike or spikelet

**Fig. 5:**
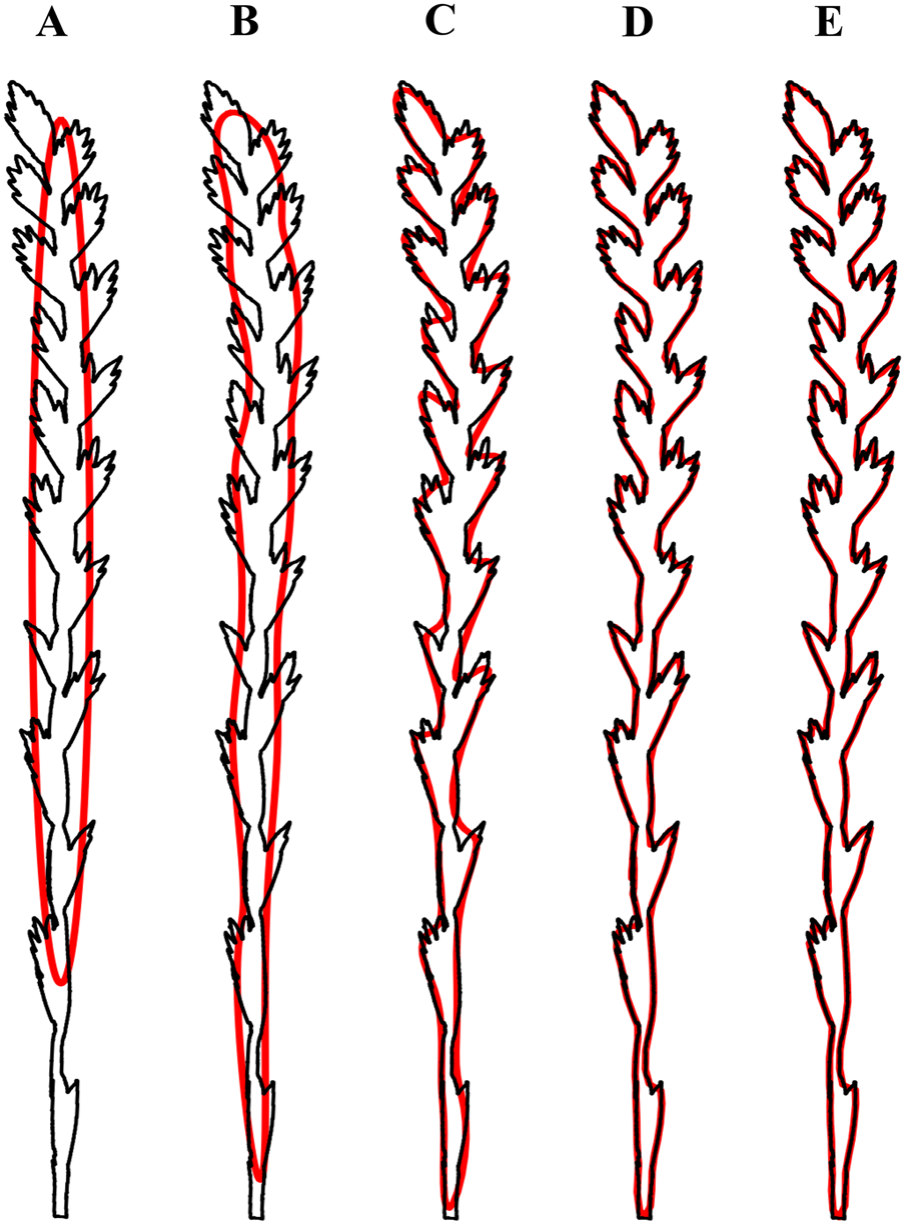
Spike shape reconstruction using Fourier series with different numbers of harmonics (n). **A** n = 1, **B** n = 10, **C** n = 30, **D** n = 81 (optimal), and **E** n = 100. The shape of the spike is outlined in black, and the outline approximation is fitted in red

The spike shape can also be quantified by deriving latent features from its contour. This is done by decomposing the outline into waves with a specified number of harmonic series using the Fourier Transform [9, 26]. The resulting Elliptical Fourier descriptors (EFDs) are the coefficients a_n_, b_n_, c_n_ and d_n,_ derived from the elliptic loci ${x}_{i}={a}_{i}coscos \theta +{b}_{i}sinsin \theta$ and ${y}_{i}={c}_{i}coscos \theta +{d}_{i}sinsin \theta$, for a point (x_i_, y_i_) with n number or harmonics [23]. The coefficients are obtained using the *CalculateEFD* function from the *spatial-efd* package [42] which is built on the *pyefd* module. To do so, we first provide a binary mask of the spike and fill any holes using the *binary_fill_holes* function from *SciPy* [41], and then execute the *findContours* function using the *RETR_CCOMP* mode and the *CHAIN_APPROX_SIMPLE* method [7] to extract the outline of the spike as paired x and y coordinates. Once obtained, the a_n_, b_n_, c_n_, and d_n_, are normalized to be insensitive to the spike’s rotation and size. The outline representation of the spike depends on the number of harmonics chosen: the more harmonics included, the more accurate the shape representation (Fig. 5). It is worth mentioning that the EFD can also be used to describe more features including symmetric (b_n_ and c_n_ harmonics) and asymmetric (a_n_ and d_n_ harmonics) sources of variance [9].

When computing the EFD, the pipeline uses the *FourierPower* function to estimate the number of harmonics needed to exceed a 0.9999 threshold Fourier power [42] as a univariate representation of the outline. This value can be used as an “optimal” number of harmonics (Fig. 5D), along with the number of spikelets per spike, and geometric descriptors such as area, perimeter, circularity, length, etc., as additional sets of shape properties from which latent variables could be derived. Unlike the original geometric variables, latent variables are orthogonal and account for maximized variation across multiple dimensions of shape [10].

### Quantifying spike color

Color is an important feature in plant inflorescence, the variation of which often provides information on maturity levels at the time of harvest. Color is a quantitative trait commonly described as qualitative because small variations in hue do not necessarily add relevant information to a biological phenomenon. However, maturity and ripening can be highly variable and arguably impossible to properly quantify at the spikelet or even plant level without high-throughput technology and data. The use of mean intensity values in a region of interest for a given color-space can be problematic when the pixel distribution is not normal. Instead of mean values per channel, SpykProps provides different percentiles across several color spaces as well as descriptors of their variation. There are currently 77 color descriptors that are output using the function *channel_percentiles* which considers all non-zero pixel values in each spike and returns the minimum, maximum, mean, standard deviation, coefficient of variation, percentiles 5, 25, 50, 75, 95, and quantile-based coefficient of variation across nine channels (R, G, B, H, S, V, L*, a*, b*). In addition, *channel_percentiles* estimates the same parameters for negative and positive values in a* and b*. The resulting dataset can be dimensionally reduced with methods such as principal component analysis, where the resulting eigenvectors could be used as latent variables to separate samples with similar color profiles.

**Fig. 6:**
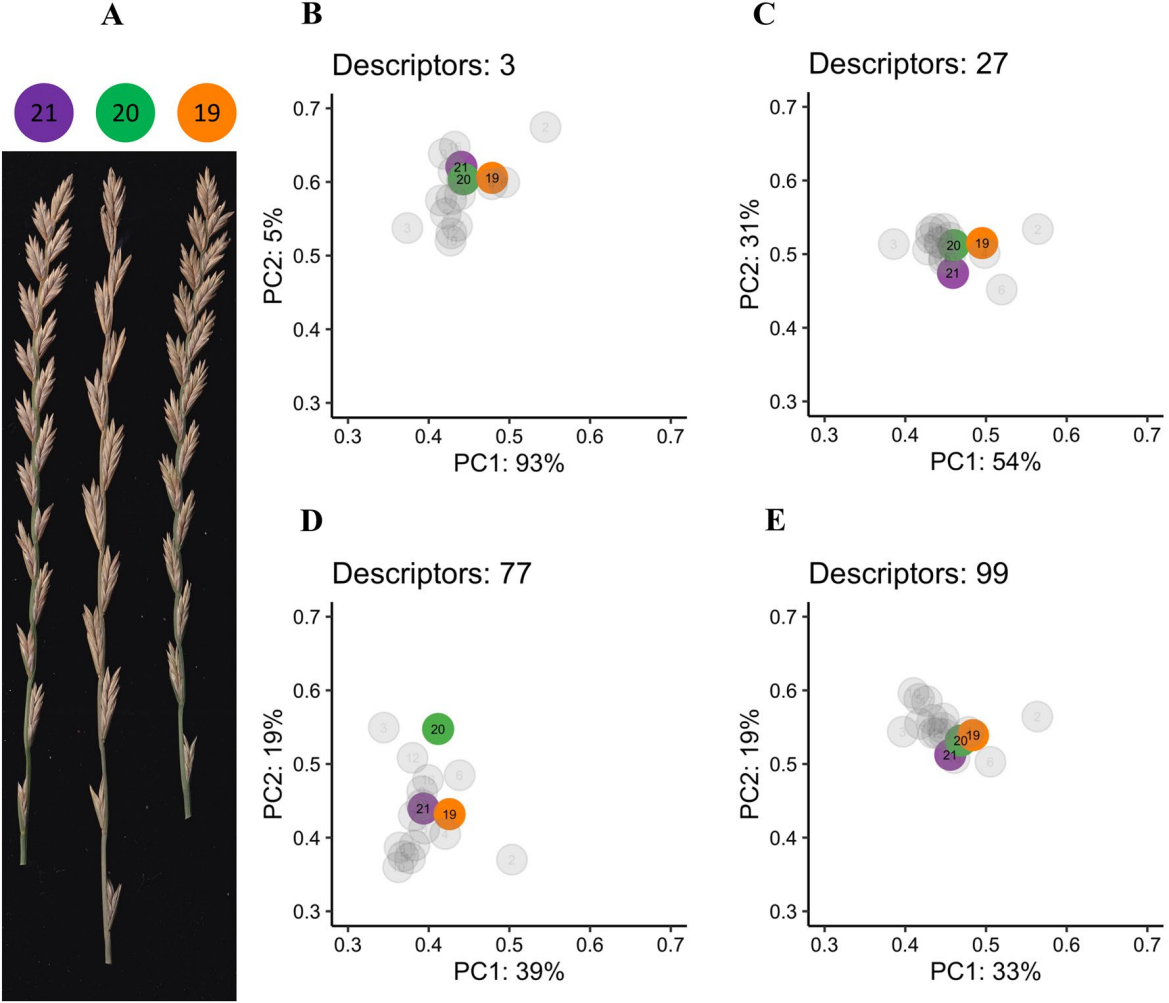
Effect of additional color descriptors on separability of spikes with similar color profile. **A** Example of three spikes with similar color profiles selected from a random image (Additional file 1: Figure S1). **B**–**E** Standardized (0–1) principal components with their percentage of variance explained, derived from various number of color descriptors for 8892 spikes encompassing 694 images. Circles and numbers correspond to a subset of all spikes in Additional file 1: Figure S2; colored circles refer to spikes in **A**. Descriptors are mean RGB values (**B**); different descriptors of the pixel frequency distribution for R, G, and B (**C**); same descriptors as in** B**, for all channels in RGB, HSV, and Lab color spaces (**D**); same descriptors as in** C** in addition to the coefficient of variation (CV) and quantile-based CV for each channel (**E**)

An example of how latent color phenotypes could help distinguish spikes with near identical color profiles is shown in Fig. 6 and Additional file 1: Fig S3. Upon projecting a subset of spikes into the first two linear combinations from variable color descriptors, we used their Euclidean distance (ED) to quantify the ability to separate spikes. In general, more descriptors tend to increase the separation between all spikes but could also introduce more noise when trying to separate a subsample of spikes. For example, using only the mean values of the RGB channels (Fig. 6B) resulted in the lowest average ED (1.92 ± 1.49) for all spikes (8892) and an average of 0.07 ± 0.04 for the spikes in Fig. 6A. Adding more descriptors to these channels (Fig. 6C) substantially increased the separation across all spikes (ED = 5.59 ± 3.82), and even more so when adding more color spaces as in Fig. 6D (ED = 7.84 ± 5.24), and more descriptors of spread as in Fig. 6E (ED = 8.2 ± 5.85). The greatest ED between spikes in Fig. 6A (0.08 ± 0.05) was achieved when using the output from SpykProps (Fig. 6D). It should be noted that this evaluation does not necessarily indicate the “best” method to use for quantifying color or a spike, but rather illustrates how using a few highly correlated variables, (i.e., mean RGB values), limits the ability to characterize and distinguish color among different spikes. Ultimately, users will need to determine the combination of color descriptors that best capture variation in color profile across their germplasm from the array of color descriptors that are output in SpykProps.

### Example application of SpykProps in perennial ryegrass breeding

**Fig. 7:**
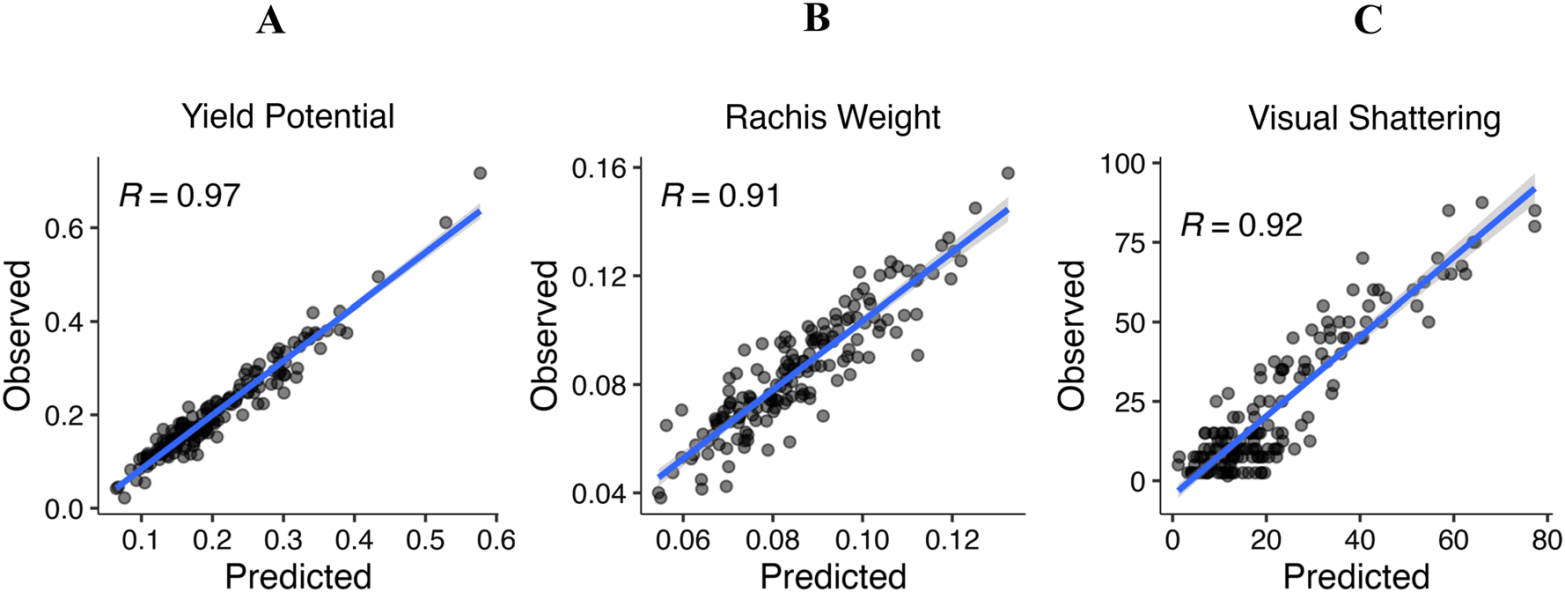
Correlation between predicted and observed agronomic values on three agronomic traits. Scatterplots with a regression line of predicted (x-axis) and observed (y-axis) trait value, with their Pearson correlation coefficient (R; p < 0.001). Gradient boosting machine (gbm) models were trained using a different set of descriptors from SpykProps

As a proof of concept to validate the applicability of our system in agronomic and breeding settings, we estimate the predictability (R^2^) of shape and color descriptors on yield-related traits measured in a perennial ryegrass nursery. Multiple machine learning algorithms showed that some spike properties are more important than others depending on the trait. Gradient boosting machine (gbm) models, in particular, consistently showed that shape and color features were good predictors of theoretical yield potential (R^2^ = 0.94), while elliptical Fourier were better for rachis weight (R^2^ = 0.83). Other traits, such as visual shattering, were best modeled using all descriptors from SpykProps (R^2^ = 0.85; Fig. 7). This suggests that the additional trait features exported from SpykProps can be used to train models to maximize accuracy and efficiency when phenotyping complex spike architectural traits associated with seed yield.

Our results suggest that latent descriptors of shape and color are relevant to the relationships affecting spike architecture and yield-related traits. While spike area was the most important feature to predict seed yield potential and shattering, latent properties of color had at least double the importance compared to the average feature (Additional file 1: Table S1). For these traits, and particularly for yield potential, descriptors of the distribution of greenness had significant importance, and so did measurements of variability such as CV and QCV. Elliptical Fourier descriptors were also relevant, especially, to predict rachis weight. Altogether, this proof of concept highlights the importance of comprehensively characterizing shape and color in understanding the relationship between spike architecture and yield related traits.

## Discussion

Our imaging platform provides advantages that distinguish it from similar systems, enabling an overall improvement in data quantity and quality. SpykProps is high throughput and fast, enabling the extraction of more than 100 traits per spike in less than three seconds, and multiple spikes at a time. This attribute is particularly crucial in scenarios where limited sample size and number of traits pose challenges to data quality. Our system minimizes human subjectivity, allowing for a comprehensive and unbiased assessment of color and shape. Unlike conventional approaches relying solely on central tendencies, our system focuses on different measures of spread and variability, providing a more comprehensive representation of color. Additionally, elliptical Fourier descriptors provide a purely quantitative definition of inflorescence shape. Such an approach has proven key to identify variation and eigenshapes associated with specific morphotypes in leaf morphology [8, 17], but are yet to be investigated in inflorescences. Importantly, our continuously-improving platform can be freely accessed from its Github repository, and can be operated without specific imaging hardware.

SpykProps has potential applications across research areas including agronomy, genetics, and developmental biology. For instance, comprehensive assessments of inflorescence morphology throughout the growing season could provide valuable insights into the dynamics affecting developmental rate and seed productivity. Similarly, our tool could be useful in comprehending disease progression, assessing its severity, and unraveling plant responses to other biotic and abiotic stresses. When coupled with environmental data, such assessments could facilitate identifying optimal timing for various agronomic management practices. Moreover, the integration of genomic resources could expand our understanding of source-sink relationships, and thus aid the development of varieties with improved resource allocation, response to biotic and abiotic stresses, and overall performance.

Given that our system is meant as a foundation for the study of inflorescence architecture through latent phenotypes, it currently has some challenges. First, SpykProps is limited to unbranched inflorescences that can be flattened out without compromising their 3D shape. For example, the system would work on perennial ryegrass, which it was developed on, and other species with similar architectures such as two-row barley (*Hordeum vulgare*) but would not currently work on 6-row barley or wheat (*Triticum aestivum*) that do not have a flat inflorescence architecture. However, researchers can overcome these challenges reconstructing their 3D-shaped samples by imaging and stitching multiple sides [38] or using proper 3D imaging hardware [39]. Second, best results require sample preparation to avoid overlapping spikes, which can increase phenotyping time. Nevertheless, SpykProps can identify overlapping spikes as outliers from the spike’s outline Fourier transform, or length, which alleviates the problem with large enough sample size. Similarly, the output data facilitates detecting outliers with anomalous colors that might influence spike segmentation; and users can also evaluate different parameters to determine adequate segmentation parameters. Lastly, unlike conventional traits, latent phenotypes are not intuitive or easy to interpret. For example, classifying a spike as “green” or with a maturity percentage, is more straightforward than calculating a score on a principal component -or a different dimensional reduction approach- from over a hundred color variables. However, the latter would account for a larger proportion of phenotypic variation, and to a greater extent, for the interaction between subtle traits that comprise the complex conventional traits.

## Conclusion

We developed SpykProps, a freely available Python-based imaging platform to measure conventional and latent phenotypes in unilateral grass inflorescences. Using perennial ryegrass spikes as a model, we showed that our high throughput phenotyping system is able to quantify spike architecture and accurately associate such morphology with yield-related traits. SpykProps has potential applications in agronomy, plant breeding, and developmental biology as demonstrated in the case example with perennial ryegrass.

## Methods

### Plant material

The spikes to develop SpykProps were obtained from perennial ryegrass plants of the variety Galactic Green that were harvested from experiments in field and greenhouse conditions [4]. The experiments sought to evaluate the effect of ethylene and gibberellin inhibitors at different rates on shattering and seed yield in perennial ryegrass. The greenhouse experiment took place in 2021 at the Minnesota Agricultural Experiment Station Plant Growth Facility in St. Paul, Minnesota, United States. The field experiment was located in Roseau, Minnesota, and comprised two locations separated by 6.5 km that were planted in 2020 as a double crop with spring wheat and harvested in 2021. Upon maturity, spikes were harvested at 0.5 mm below the last spikelet, bagged in paper bags, and stored for further processing. Variation across environments and inhibitor rates provided a wide range of size and color within and among spikes.

### Data collection

A total of 166 images were obtained by carefully placing the dry spikes on a CanoScan Lide 300 flatbed scanner at 600 dpi and 107 MB in disk. All images were 7016 pixels in height by 5104 pixels width and contained between 5 and 23 spikes. We used a black velvet as a background for the images which reduced light reflectance and potential artifacts in the images. The data collected from the pipeline was obtained using a MacBook Pro 2021 with an Apple M1 Pro chip, a 16 GB memory, and running macOS Monterrey.

### Imaging pipeline

This pipeline was developed using Python to segment spike and spikelets before extracting features describing variation in morphology. The system includes functions to visualize, compare, and extract data for a single spike, but it is meant to be used on a set of images containing multiple spikes. Ideally, such images contain inflorescences of the same plots, replication, or plant. The most complete datasets are obtained by running the *SpykBatch.py* function, which only requires the path to the images in a Python list. Once finished, the function returns different datasets containing spikes and spikelets color and shape descriptors. Color data was generated in Python using the spike's binary mask and its RGB version to characterize the pixel distribution across color-spaces and channels. In addition, we developed R functions to generate coefficients of variation (CV) and quartile-based coefficients of variation (QCV) for the color data. The shape data comprises elliptical Fourier descriptors (EFD), as well as geometric properties extracted from the spike's binary mask using the *regionprops* function (Van der Walt et al., n.d.), both available for Python.When computing the EFD, the pipeline uses the *FourierPower* function to estimate the number of harmonics needed to exceed a 0.9999 threshold Fourier power [42], unless they are specified by the user.

### Validation and models

We developed multiple machine learning models on SpyProps data from a first-year production nursery evaluated for seed shattering and other seed yield traits. The nursery was located in the Turfgrass Research, Outreach and Education Center at the University of Minnesota, and comprised 20 different maternal clones and their half-sib families (Additional file 1: Fig S4), replicated across three blocks. The traits were collected from approximately 10 spikes per plot that were harvested and imaged as described above. In summary, each sample was first given average percentage scores per spike for visual shattering estimate (VSE) at harvest and for total seed retention (RET) after induced detachment with a wrist action shaker. Next, we weighed the seed detached before and after shaking (DBS and DAS, respectively), hand-threshed the spikes to weigh the seed retained before and after shaking (RBS and RAS, respectively), and derived the rachis weight (RW) and theoretical yield potential (TYP) from the measurements [4]. In addition, an averaged developmental rate score (DEV) was also given based on a phenological scale for cool-season perennial grasses that has previously been described [18]. The descriptive statistics for agronomic traits can be found on Additional file 2: Table S2. Lastly, we simulated a normally distributed trait with mean zero and standard deviation one, as a control to compare predictions.

Combinations of shape and color descriptors were derived from the parents and offspring and used to train the models with data from 70% of the families. Because spike shape and particularly curvature was affected by storing bags, we removed spikes with curvature greater than 5.5 standard deviations above the mean). In some plots, this induced the need for sampling from the imaging data to match the number of processed spikes in the agronomic data. We filtered out near-zero-variance predictors as well as those with a Pearson’s correlation coefficient greater than 0.85, and scale and center the remaining variables before training. All models were trained using the Caret package [24], cross-validated using leave-one-out (LOOCV) method, and their performance was evaluated based on their root mean squared error (RMSE), mean absolute error (MAE), and the Pearson correlation coefficient (R) between the predicted and observed values for both the training and testing datasets (Additional file 3: Table S3). The models included: partial least squares (*pls*), ridge (*ridge*), least absolute shrinkage and selection operator (*lasso*), and principal component regression (*pcr*), elastic net (*enet*), gradient boosting machine (*gbm*), support vector machine radial (*svmRadial*) and lineal (*svmLinear*), and random forest (*rf*). Tuning parameters were set based on Caret's default values, which included: up to 30 eigenvectors for *pls* and *pcr*; C = 1 for *svmLinear* and *svmRadial*, with the latter including sigma = 0.1; lambda = 0.1 for *ridge* and *enet*; similar to *lasso*, *enet* included fraction = 0.1; finally, *gbm* was evaluated with different values for tree number (50, 100, and 150), interaction depth (1, 5, 9), but fixed shrinkage (0.1) and n.minobsinnode (10). The model with the highest R^2^ was kept when multiple parameters were tested.

### Supplementary Information

**Additional file 1: Figure S1.** Example of an RGB image with segmented and numbered spikes. **Figure S2.** Spikelet detection with different values for minimum distance. When running the program on a batch of spikes, users should consider the proper minimum distance based on the average number of misdetections, and whether they can be easily filtered as outliers, for example, based on size or shape. **Figure S3.** Pixel distribution across different descriptors of greenness for three spikes with seemingly alike color profiles. Boxes in density plots indicate the corresponding mean pixel intensity and standard deviation for the spikes in Figure 6 across three channels from different color spaces. Hue in the heatmap indicates the standardized value that each spike has for all color descriptors studied in this project. This shows that the similarity between spikes depends on the descriptor and channels that are being considered. **Figure S4.** Number of observations for agronomic data by type of germplasm across 20 families. **Table S1.** Top five most important features for predicting yield related traits using SpykProps.

**Additional file 2: Table S2.** Descriptive statistics for modeled agronomic traits.

**Additional file 3: Table S3.** Results of all machine learning models tested.

## Data Availability

SpykProps, along with all its Python components is freely available at https://github.com/joanmanbar/SpykProps. All the original and processed images, along with the data files and code to analyze them, can be accessed through the Data Repository for University of Minnesota (DRUM) at https://hdl.handle.net/11299/256105.

## Abbreviations

CV: Coefficient of variation
DAS: Detached after shaking
DBS: Detached before shaking
DEV: Developmental stage
DPI: Dots per inch
ED: Euclidean distance
EFD: Elliptical Fourier descriptor
ENET: Elastic net
GB: Gigabytes
GBM: Gradient boosting machine
HSV: Hue, saturation, value
HTP: High throughput phenotyping
LASSO: Least absolute shrinkage and selection operator
MB: Megabytes
PCA: Principal component analysis
PCR: Principal component regression
PLS: Partial least squares
QCV: Quantile-based coefficient of variation
RAS: Retained after shaking
RBS: Retained before shaking
RET: Seed retention
RF: Random forest
RGB: Red, green, blue
ROI: Region of interest
RMSE: Root mean square error
RW: Rachis weight
SVM: Support vector machine
TYP: Theoretical yield potential
VSE: Visual shattering estimate
2D: Two dimensional image
3D: Three dimensional image
