# Supplementary material for: SpykProps: an imaging pipeline to quantify architecture in unilateral grass inflorescences
Source: Plant Methods. 2023 Nov 13;19:125. doi: 10.1186/s13007-023-01104-z (PMC10644492; doi:10.1186/s13007-023-01104-z)
Supplement: Supplementary file 1 — Additional file 1: Figure S1. Example of an RGB image with segmented and numbered spikes. Figure S2. Spikelet detection with different values for minimum distance. When running the program on a batch of spikes, users should consider the proper minimum distance based on the average number of misdetections, and whether they can be easily filtered as outliers, for example, based on size or shape. Figure S3. Pixel distribution across different descriptors of greenness for three spikes with seemingly alike color profiles. Boxes in density plots indicate the corresponding mean pixel intensity and standard deviation for the spikes in Figure 6 across three channels from different color spaces. Hue in the heatmap indicates the standardized value that each spike has for all color descriptors studied in this project. This shows that the similarity between spikes depends on the descriptor and channels that are being considered. Figure S4. Number of observations for agronomic data by type of germplasm across 20 families. Table S1. Top five most important features for predicting yield related traits using SpykProps. [file 13007_2023_1104_MOESM1_ESM.pdf]

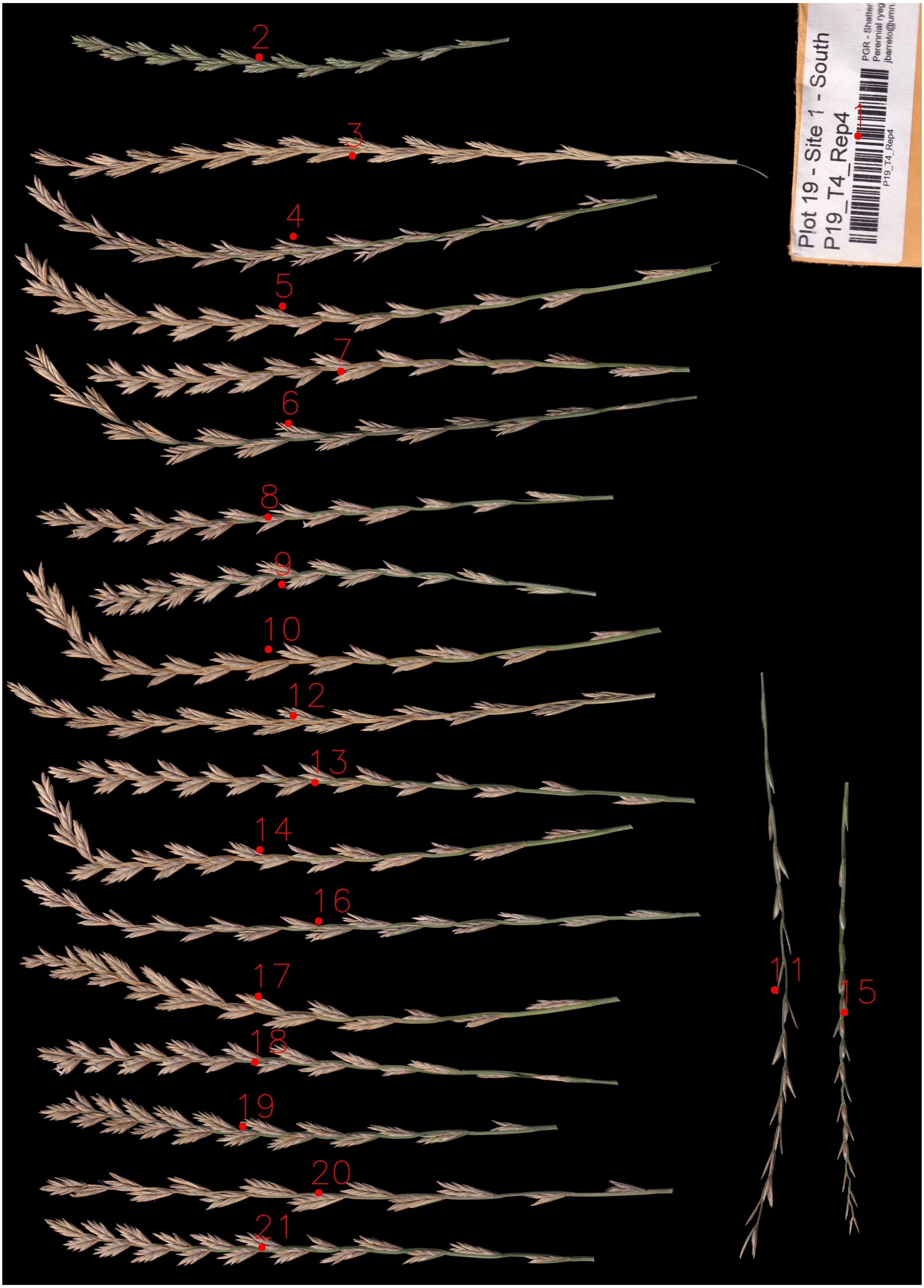

Figure S1. Example of an RGB image with segmented and numbered spikes.

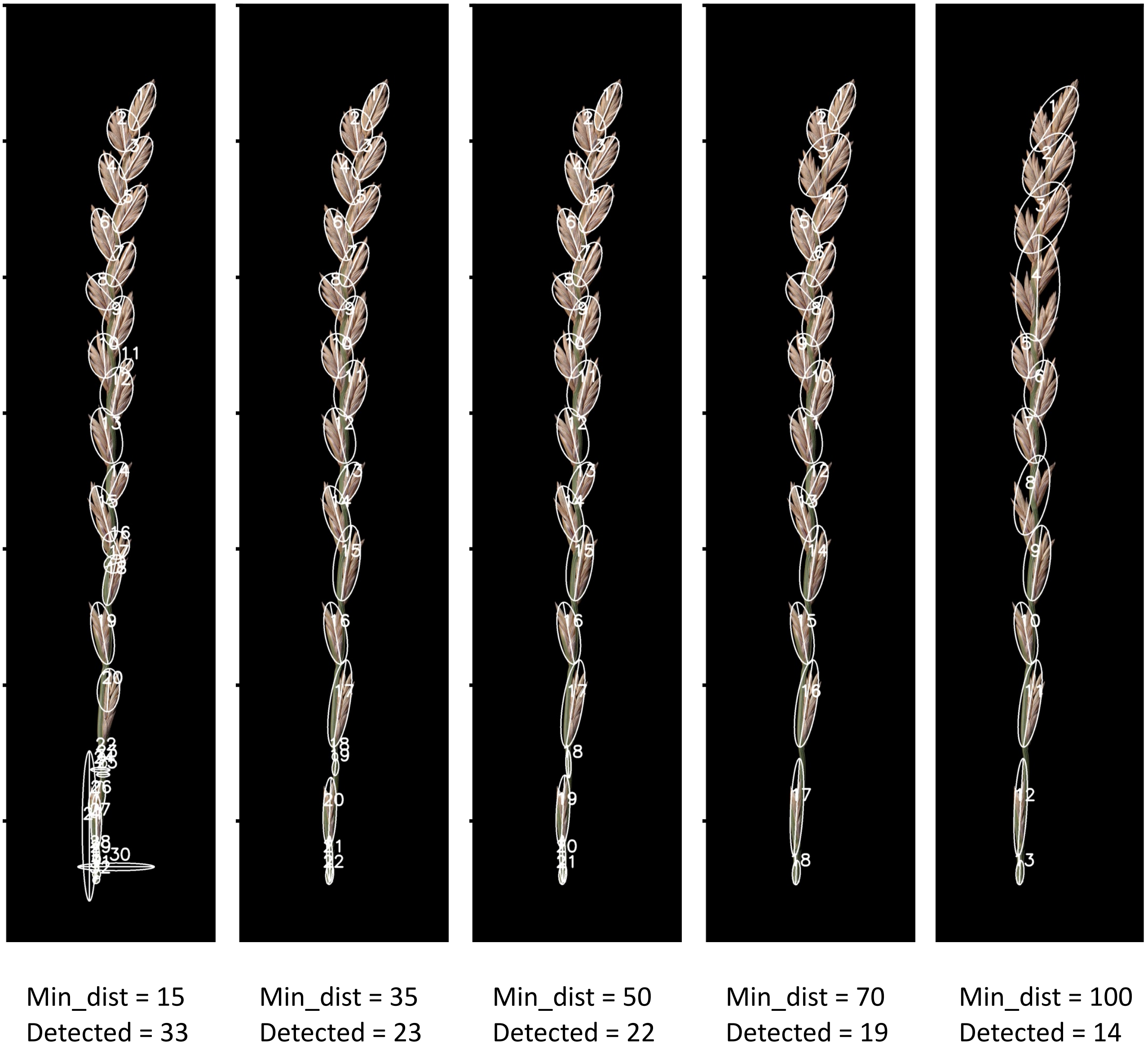

Figure S2. Spikelet detection with different values for minimum distance. When running the program on a batch of spikes, users should consider the proper minimum distance based on the average number of misdetections, and whether they can be easily filtered as outliers, for example, based on size or shape.

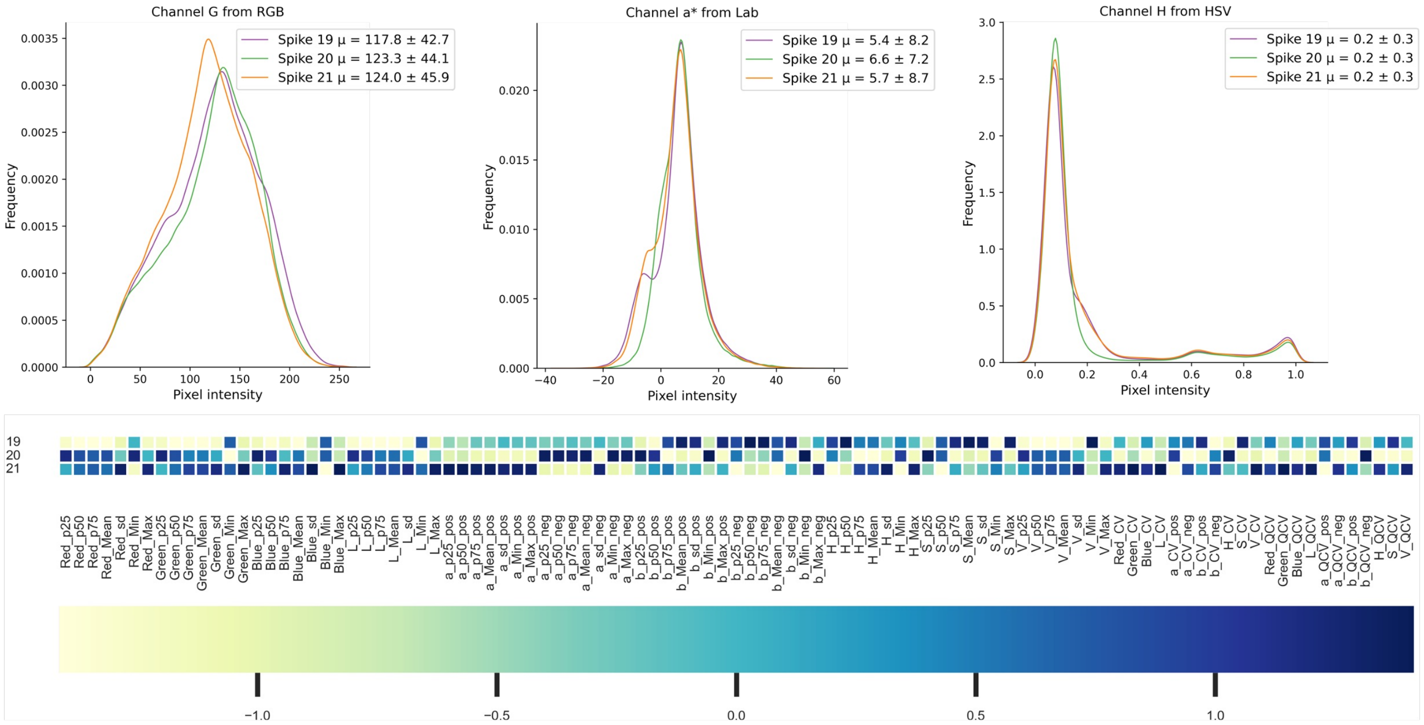

Figure S3. Pixel distribution across different descriptors of greenness for three spikes with seemingly alike color profiles. Boxes in density plots indicate the corresponding mean pixel intensity and standard deviation for the spikes in Figure 6 across three channels from different color spaces. Hue in the heatmap indicates the standardized value that each spike has for all color descriptors studied in this project. This shows that the similarity between spikes depends on the descriptor and channels that are being considered.

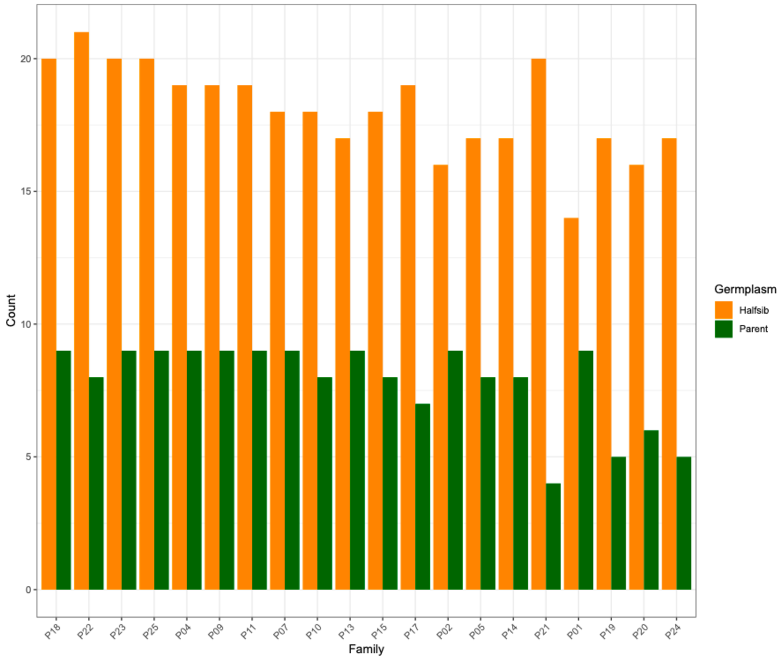

Figure S4. Number of observations for agronomic data by type of germplasm across 20 families.

Supplemental Table S1. Top five most important features for predicting yield related traits using SpykProps.

Trait	Dataset	Feature	Importance (%)
Theoretical seed yield potential	Shape and color (137 features)	Area	23.5
		a_Min_pos[a]	7.4
		a_QCV_pos	4.9
		b_Min_pos	4.4
		a_CV_neg	3.6
		**All features average**	**1.8**
Rachis weight	Elliptical Fourier and color descriptors (250 features)	H_p25[b]	3.7
		Bn24[c]	2.5
		An3	2.3
		Bn13	2.2
		Green_Min	2.1
		**All features average**	**0.6**
Visual shattering estimate	Color, shape, and elliptical Fourier descriptors (256 features)	Area	11.8
		Dn1	6.7
		Green_Min	4.5
		Solidity	4.3
		Blue_max	2.6
		**All features average**	**0.6**

[a] Features containing underscores in their names indicate the color channel, followed by the metric, and whether they are positive or negative. For example, a_Min_pos refers to minimum positive value in the channel a.
[b] A lowercase p followed by a number, indicates a percentile for the given channel.
[c] Letters A, B, C, or D, followed by an n, indicate coefficients from the Fourier transformation (see Figure 5).
